# Components of the Fiber Diet in the Prevention and Treatment of IBD—An Update

**DOI:** 10.3390/nu15010162

**Published:** 2022-12-29

**Authors:** Katarzyna Ferenc, Sara Jarmakiewicz-Czaja, Rafał Filip

**Affiliations:** 1 Institute of Medicine, Medical College of Rzeszow University, 35-959 Rzeszow, Poland; 2 Institute of Health Sciences, Medical College of Rzeszow University, 35-959 Rzeszow, Poland; 3 Department of Gastroenterology with IBD Unit, Clinical Hospital No. 2, 35-301 Rzeszow, Poland

**Keywords:** Crohn’s diseases, dietary fiber, inflammatory bowel diseases, nutrition, ulcerative colitis

## Abstract

Inflammatory bowel disease (IBD) is a group of diseases with a chronic course, characterized by periods of exacerbation and remission. One of the elements that could potentially predispose to IBD is, among others, a low-fiber diet. Dietary fiber has many functions in the human body. One of the most important is its influence on the composition of the intestinal microflora. Intestinal dysbiosis, as well as chronic inflammation that occurs, are hallmarks of IBD. Individual components of dietary fiber, such as β-glucan, pectin, starch, inulin, fructooligosaccharides, or hemicellulose, can significantly affect preventive effects in IBD by modulating the composition of the intestinal microbiota or sealing the intestinal barrier, among other things. The main objective of the review is to provide information on the effects of individual fiber components of the diet on the risk of IBD, including, among other things, altering the composition of the intestinal microbiota.

## 1. Introduction

Inflammatory bowel diseases (IBDs) are a group of diseases with a chronic course, characterized by periods of exacerbation and remission of the disease. The etiology of IBD is not fully understood; however, many researchers point to a predisposition to the onset of the disease with the existence of certain genetic, environmental, immunological, and microbiological factors [1,2,3]. IBD can occur in both men and women of all ages. Western lifestyles are causing an increase in the incidence of IBD year after year. Researchers predict that their prevalence will increase significantly in the next few years [4].

A potentially predisposing component to IBD is a Western-type diet, which is, among other things, low in dietary fiber.

Dietary fiber is a broad concept, so different classifications are used to describe it. A division by origin, physicochemical properties, and chemical composition is used [5]. Depending on the solubility of dietary fiber in water, it can be classified as soluble (SDF) and insoluble (IDF). These groups differ in their functionality and mode of action after ingestion [6]. Soluble fiber includes fructooligosaccharides, galactooligosaccharides, pectins, β-glucans and inulin [7]. The second group includes cellulose, hemicellulose, and lignins, among others [8]. Dietary fiber is found in various proportions and in many foods, such as vegetables, fruits, pulses, nuts, seeds, and cereals. However, not all types of fiber are found in the same food groups; pectin is more abundant in fruits and some types of vegetables, and β-glucans are found in cereals [9]. Starchy foods that contain resistant starch include pulses, cereals, and potatoes [10]. Insoluble fiber functions as a means of increasing fecal weight and reducing intestinal transit time, which consequently contributes to relief from constipation [11]. Both soluble and insoluble fibers are indigestible. However, soluble in the presence of water can be quickly and easily fermented by intestinal bacteria into products that act favorably on the intestinal microbiome, mainly short-chain fatty acids (SCFAs) [12]. Therefore, ultimately, it may have some prebiotic functions, but it may also positively impact health by reducing the risk of gastrointestinal diseases, such as irritable bowel syndrome (IBS), inflammatory bowel disease (IBD), or constipation [13].

β-glucans are a natural group of polysaccharides consisting of D-glucose monomer units linked by β-glycosidic bonds (1,3, 1,4, 1,6). β-glucans can be found in yeast, fungi, some bacteria, seaweed, and cereals, mainly in oats and barley [14]. The diversity and biological activity of these compounds depend on their molecular structure, the conformation of each polymer, and their solubility [15]. β-glucans from oats have actions typical of dietary fiber through which they improve metabolic health parameters, that is, cholesterol and glucose levels [16,17]. β-glucans from yeast and fungi act as immunomodulators. These compounds work by activating the immune system by initiating the inflammatory process, increasing the response to infections, and through antitumor effects [18]. β-glucans appear to be an interesting option to support drug therapy in various diseases. In this review, we describe current knowledge on the effects of dietary fiber components with special emphasis on disruting the composition of the intestinal microbiota.

## 2. Influence of Fiber on the Intestinal Microbiota

The intestinal microbiota is all the microorganisms that inhabit the intestines. They include bacteria, viruses, fungi, archaeons, and selected unicellular eukaryotes. On the contrary, the definition of the gut microbiome is the entire collection of genes from microorganisms that reside in the intestines [19]. The human gut microbiome population includes more than 1000 microbial species. The most numerous species are *Bacteroidetes* and *Firmicutes* and slightly less numerous are *Proteobacteria*, *Actinobacteria*, *Verrucomicrobia*, *Fusobacteria*, *Cyanobacteria*, and others [20]. Most bacteria that live in the intestines are anaerobic microorganisms. The presence of aerobic bacteria has been observed primarily in the cecum.

There are several factors that modulate the composition of the intestinal microbiota. One factor is the use of antibiotics. These are prescription drugs that are often given to children from the first days of life. Due to the dynamic development of the intestinal microbiota in children, it is particularly sensitive to antibiotics. The use of these drugs can affect a decrease in *Bifidobacteriaceae* and *Lactobacillales* spp., while it can predispose to an increase in *Enterobacteriaceae* [21]. In their work, Ianiro et al. point out that the effect of antibiotics on the gut microbiota depends on the type of drug, its dose, and the route of administration, as well as factors that are directly related to humans. However, antibiotic administration can also have a eubiotic effect on the host, that is, it can stimulate the growth of beneficial bacteria [22]. However, the use of this group of drugs is most often associated with the appearance of intestinal dysbiosis, i.e., abnormalities in the composition/function of intestinal microorganisms, which can lead to the development of certain diseases or the appearance of exacerbations [23]. The adverse effects of inadequate antibiotic therapy can also include drug resistance, and thus the development of pathogenic microorganisms and a reduction in the commensal microbiota and its diversity [24].

Another factor that modulates gut microbiota is host genetics [25,26]. The heritability of the microbiome ranges from 2% to 8%. However, the authors of some studies show that environmental factors outweigh the genetic factors responsible for the composition of the host microbiota [27]. In their study, Matsumoto et al. showed, excluding genetic factors, that individual dietary components affect certain bacterial species [28]. Polyphenols, proteins, fats, and dietary fiber are essential components of the metabolic pathways of the intestinal microbiota [29].

There are also scientific reports that highlight the effects of specific types of diet on the intestinal microbiota, so, for example, a vegan diet has a beneficial effect in increasing the number of beneficial microorganisms, while following a low FODMAP diet changes the ratio of *Firmicutes* to *Bacteroidetes* and decreases the number of *Bifidobacterium* [30,31]. Other researchers have also indicated the beneficial effects of a plant-based diet on the gut microbiota [32,33]. Reddel et al. point out that when using low-FODMAP, gluten-free, or ketogenic diets in various pathological conditions, supplementation with selected nutrients should be considered, as these diets can significantly exacerbate already existing changes in the gut microbiota [34]. Other authors also point to the effect of age on the composition of the intestinal microbiota [35,36].

**Figure 1:**
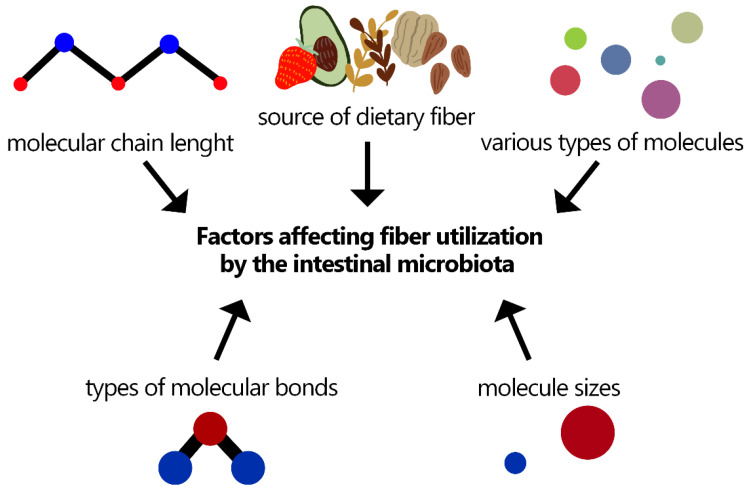
Factors affecting fiber utilization by the intestinal microbiota [37].

Dietary fiber has a variety of functions in the human body. One of the most important aspects is its effect on the intestinal microbiome and consequently on the prevention of the occurrence of certain diseases. The utilization of dietary fiber by the intestinal microbiota depends on several factors (Figure 1) [37].

Dietary fiber, which is only available to the intestinal microbiota, is called MAC (microbiota-accessible carbohydrates). It is mainly a source of energy for the intestinal microbiota. MAC is lower when following a Western-type diet, that is, one with low amounts of dietary fiber. Low MAC can reduce the abundance of some commensal bacterial taxa, which is detrimental to the host [38]. In their work, Usuda et al. indicate that decreased MAC can lead to increased intestinal permeability and the induction of colitis [39]. The reason for this may be a decrease in the production of the receptor agonists of glucagon-like peptide 1 (GLP-1) and glucagon-like peptide 2 (GLP-2), which are required for intestinal regeneration after the appearance of mucosal inflammation [40]. Increasing the MAC may favorably influence the increase in the abundance of *Bacteroides thetaiotaomicron*, *Bifidobacterium* spp. [39]. The failure to include dietary fiber can lead to an increase in *Clostridium* spp., *mucinophilic bacteria*, and thus increase the risk of inflammation [41]. In turn, a higher content of this dietary component increases the synthesis of SCFA (short chain fatty acids). Myhrstad et al. indicate that this is due to an increase in bacteria such as *Ruminococcus*, *Lachnospira*, *Akkermannsia*, *Bifidobacterium*, *Lactobacillus*, and *Roseburia* [42]. Furthermore, Angelis et al. indicate that it can also reduce the secretion of pro-inflammatory substances, such as trimethylamine N-oxide (low molecular weight uremic toxin), indoxyl sulfate (metabolic product of tryptophan breakdown), and p-cresyl sulfate (product of metabolism of tyrosine and phenylalanine by intestinal bacteria) [29].

Adequate mucus production is essential to maintain complete intestinal health. Dietary fiber has the ability to stimulate the intestinal epithelium to secrete mucus through a mechanical action on the epithelium. Acetate, butyrate, and propionate, which belong to the SCFA group, show the ability to regulate pH in the intestinal lumen and are essential for the supply of energy to enterocytes [43]. In addition, they affect the production of mucus in the intestines. Propionate can be synthesized through three different pathways by intestinal bacteria that reside in the human gut: the acrylate, propanediol, and succinate pathways, with succinate being the most common. Butyrate is synthesized from two molecules, the latter of which is CoA-transferase in the presence of acetate, which is essential for efficient synthesis of the compound. On the other hand, acetate production occurs from acetyl-coenzyme A through acetyl-CoA to produce the substance [44,45,46]. Increasing the amount of dietary fiber in the daily diet predisposes to increased amounts of *Lactobacillus* spp. and *Bifidobacterium*. This is mainly due to an increase in the intake of fructans and galactooligosaccharides [47]. In a study by Fischer et al. in animal models, it was shown that indirectly through adequate fiber, the intestinal microflora containing A. finegoldii, among others, showed that increased intestinal expression of IL-22. IL-22 is responsible, among other things, for maintaining adequate intestinal barrier function due to the separation of microorganisms from the intestinal epithelium [48]. A high-fiber diet also reduces pro-inflammatory cytokines [49].

The soluble fraction of dietary fiber is used by intestinal microbes to obtain energy through its breakdown into oligosaccharides/monosaccharides [50]. The soluble fraction of dietary fiber includes β-glucans, which are increasingly being studied for their effects on the intestinal microbiome [51]. These polymers composed of D-glucose linked by a β-glycosidic bond show the ability to decrease *Enterobacteriaceae*, while increasing *Bifidobacteria* and *Lactobacilli* [50]. In their study, Wang et al. indicate that β-glucan has the ability to modulate intestinal microflora; however, this depends on its molecular weight. The authors note that for microflora modulation, the use would be to introduce a compound of high molecular weight, since supplementation with 3 g of high-molecular-weight β-glucan increased *Bacteroidetes*, while it decreased *Firmicutes*, although diets with 3 g of supplementation per day and 5 g of low molecular weight β-glucan did not change the composition of the intestinal microflora [52]. Carlson et al. analyzed the fermentation of various prebiotics, including β-glucan. They showed that β-glucan and oatmeal containing 28% β-glucan had a significant increase in propionate concentration compared to other compounds they studied (Xyloligosaccharide (XOS) and Inulin, a mixture of dried chicory root containing inulin, pectin, hemi/cellulose) [53]. In another study, the researchers compared the fecal microbiota and metabolomics after a 2-month intervention using 3 g of barley β-glucans. They showed a significant increase in SCFA such as acetic, 2-methylpropanoic, propionic, and butyric acids, suggesting modulation of the composition and metabolic pathways of the intestinal microbiota [54].

Intestinal dysbiosis is one of the features attributed to IBD. It is characterized by a decrease in microbial diversity, an increase in unfavorable pathogenic bacteria, and a decrease in beneficial anaerobic bacteria [55]. Patients with IBD patients show a decrease in the number of bacteria, mainly *Firmicutes*, while an increase in the population of *Proteobacteria*. There is a lack of information on whether intestinal dysbiosis in IBD is the cause or one of the consequences of the disease [56]. Intestinal dysbiosis and the associated loss of bacterial diversity can lead to the loss of key functions of the normal intestinal barrier. The result can be a dysregulation of the immune system. These dysfunctions can potentially cause inflammation and a stimulated immune response. As a result, they can contribute to IBD [57]. The fermentation of dietary fiber promotes the formation of short-chain fatty acids (SCFA). These acids have anti-inflammatory effects that protect the intestinal epithelium. One of the bacteria that produce SCFA is *F*. *prauznitzii*. Interestingly, a study shows that a lower percentage of these bacteria in the ileum of CD patients is associated with endoscopic recurrence after a period of 6 months. Sokol et al. propose the use of F prausnitzii as a potential probiotic for the treatment of CD [58]. Chiba reports that the amount of *F*. *prausnitzii* in CD patients is significantly lower than in healthy individuals. He suggests that a diet rich in fiber does not harm but supports and benefits CD patients [59]. The breakdown of fiber in SCFA by intestinal microbes contributes to a favorable regulation of the intestinal microbiome [60]. Interestingly, it seems that CD patients have a much more pronounced intestinal dysbiosis compared to UC. Lower microbial diversity and poorer stability are observed. Even in the context of the microbiome, CD and UC are distinct disease entities at the microbiome level [61]. UC patients have been found to have a reduced number of butyrate-producing bacteria *R. intestinalis* and *F. prausnitzii*. This appears to be strongly correlated with reduced SCFA in patients with UC [62]. Patients with CD showed an increase in *Ruminococcus gnavus* and a decrease in *F. prausnitzii*, *Bifidobacterium adolescentis*, *Dialister invisus*, and other bacteria that produce butyrates in stool samples. These findings encourage researchers to further investigate the use of SCFA as a complementary treatment for patients with IBD [63]. Currently, the analysis of the gut microbiota has led researchers to the possibility of using microflora transplants as a therapeutic modality for patients with IBD [64].

## 3. Link between Dietary Fiber and IBD

The development of inflammatory bowel disease (IBD) is believed to be influenced by environmental factors, genetic conditions, a weak immune system, and changes in the microbiota. Recently, there has been increasing interest in the dietary factor, which plays an important role in the etiopathogenesis and course of the disease. It is important that a properly selected diet can reduce the risk of developing IBD. Controlling the patient’s diet allows the prolongation of remission, which consequently increases the patient’s quality of life [65]. The pro-inflammatory Western diet found in highly developed countries can increase the risk of inflammatory bowel disease by promoting intestinal dysbiosis, disrupting the immune system, and compromising intestinal permeability. Therefore, many researchers have investigated the diet and its specific nutrients as a potential therapeutic agent for patients with IBD [66].

A crossover study was recently published that included patients with ulcerative colitis in remission or mild exacerbation. The purpose of the study was to determine the effect of a low-fat, high-fiber diet compared to the typical American diet (SAD) and the improved version of the American diet (iSAD). Patients on a low-fat, high-fiber diet (LFD) showed a tendency to improve their microbiota due to an increase in *Bacteroidetes*, *F prausnitzii*, and *Prevotella* and a decrease in *Actinobacteria*. Furthermore, there was a tendency to decrease the CRP compared to the baseline result. Interestingly, LFD, as well as iSAD, significantly improved the clinical symptoms of the patients. Despite the presence of high-fat products and the high prevalence of meat products in iSAD, the results improved. This is attributed to an increase in the intake of fiber and monounsaturated fatty acids in the diet or a placebo effect due to the perception of care of the diet caterer. Importantly, this study shows that patients with UC in remission and mild exacerbation can be treated with a high-fiber diet without side effects and with the possibility of favorable results [67].

Chronic inflammation in the intestine is a hallmark of inflammatory bowel disease. Reducing inflammation and completely treating the affected area is undoubtedly a goal of physicians but also a huge challenge [68]. A published review by Swann et al. suggests that high fiber intake may be beneficial in preventing and reducing inflammation. This probably occurs by modifying pH and intestinal permeability [69]. Patients with IBD are found to have an increased risk of cancer, which accounts for about 10 to 15% of deaths annually. This occurs due to the chronic inflammation of the gut and impaired immune function [70]. There are studies showing that a diet rich in dietary fiber may have a preventive effect on the appearance of cancer [71,72]. Recent studies have shown that high fiber intake, especially cereals, is correlated with a reduced risk of developing and the progression of colorectal cancer [73,74,75].

### 3.1. β-Glucan

Liu et al. tested the effect of β-glucan from oats on intestinal inflammation induced by dextran sulfate sodium (DSS) in animal models. They showed that the administration of β-glucan was able to reduce the expression of IL-1β, TNF-α, and IL-6 and decrease the activity of myeloperoxidase (MPO) and malondialdehyde (MDA), thus reducing the severity of inflammation. In addition, the authors noted a beneficial effect in reducing the activity of clinical symptoms [76]. On the other hand, Bai et al. indicated that β-glucan treatment increases the concentration of butyrate, acetate, and propionate, which has a beneficial effect on the metabolism of commensal microorganisms, thus promoting anti-inflammatory effects [77]. In addition, in their study, Chen et al. showed that β-glucan derived from mountain barley can increase the transcription of genes responsible for encoding ZO-1, occludin, mucin2 (MUC2), and claudin-1, thus enhancing the intestinal barrier in a mouse model of DSS-induced UC [78]. Furthermore, many authors of the study also point to the important role of β-glucan in modulating the intestinal microflora in the presence of other disorders, such as obesity [79,80,81].

Faghfoori et al. showed in their study that β-glucan from barley in patients with ulcerative colitis (UC) causes a reduction in C-reactive protein (CRP) levels. Improvements in clinical symptoms were also observed compared to the control group. Importantly, no side effects were found in β-glucan intake [82]. The mechanism of this effect appears to be the production of butyrate by β-glucan from barley, which may alleviate inflammation in patients with UC. However, more research is needed on this issue [83]. As a result, diet fiber can regulate intestinal mechanisms and improve patients’ quality of life. One such mechanism is the regulation of bowel movements. This is acheived with the help of water absorption by fiber, stool softening, and the formation of adequate fecal mass. The result is, among other things, the prevention of hemorrhoids and diverticulitis [60]. An adequate amount of dietary fiber in the diet and regular intake can help modulate digestion. This has the effect of reducing intestinal transit time, which contributes to the control of carbohydrate and lipid metabolism [84]. This is found especially in the effects of high-viscosity fibers (e.g., gelatinous β-glucan, plantain) [85]. In recent years, a study was conducted involving IBD patients with IBS symptoms. The effect of a mixture of β-glucan, inositol, and digestive enzymes on gastrointestinal symptoms was studied in patients taking mesalazine. Patients in the mixed supplemented group compared to the control group (mesalazine only) showed a reduction in abdominal pain and bloating but also an improvement in their general condition. Spagnuolo et al. suggest that the improvement in gastrointestinal symptoms may be due to the anti-inflammatory effects of β-glucan. β-glucan reduces the production of pro-inflammatory cytokines, such as IL-10, IL-12, and TNF-α. The improvement in side effects was certainly enhanced by the prebiotic effect of fiber through its effect on modulating intestinal microflora. A limitation of this study is the small number of subjects (*n* = 23) [86]. In addition to the beneficial effects of β-glucan on the gut, it can also have positive effects on organs. An example is the beneficial effect of β-glucan, extracted from oats, on gastritis by, among other things, reducing mucosal damage [87].

### 3.2. Pectin

A diet enriched with orange pectin in mice with experimentally induced UC may exert additional protective effects against the development of the disease. This is due in part to the inhibition of reactive oxygen species (ROS) production by pectin-derived oligosaccharides. This may inhibit Th17 accumulation, which is often found in the colon of UC patients. Despite this, it appears that Th17 suppression is insufficient to alleviate symptoms of colitis, if only because citrus pectin applied to mice showed an exacerbation of the disease compared to a control sample. It was also found that mice fed orange pectin had an increase in Th1 in the colon. Th1 does not appear to inhibit the anti-inflammatory effects of SCFA [88]. Another study shows that pectin reduces the severity of colitis in mice [89].

In another prospective human study (Nurses Health Study), Ananthakrishnan et al. showed that the consumption of fruits rich in dietary fiber may be a protective factor in the appearance of CD, while it had no effect on the risk of UC [90]. However, in the Nurses Health Study II, Ananthakrishnan et al. found that a diet high in dietary fiber and fish during highschool can have a protective effect against the appearance of CD. Such relationships were not confirmed for UC [91]. In a review, Wu et al. demonstrated the potential mechanisms of pectins through which they exert their protective effects against IBD. They mention, among other things, the positive effects on intestinal dysbiosis, the regulation of the immune system, and the inhibition of pathogen adhesion [92]. Pectins are also used in studies as drug carriers [93,94].

### 3.3. Starch

Another example of a substance that shows beneficial effects on IBD is resistant starch (RS), which may be associated with reduced mucosal damage.

The study presented by Shinde et al. is an observation of the effects of resistant starch derived from green bananas and the spores of the probiotic *Bacillus* coagulans MTCC5856 on colitis in mice induced by DSS (sodium sulfate). The authors showed that both compounds showed beneficial effects in reducing colon inflammation [86]. In another study involving animal models, the authors fed mice a diet rich in wheat flour and showed that they had milder colitis [88]. On the contrary, Zhang et al. studied the effects of type 2 resistant starch (RS2) on inflammation and intestinal permeability in mice that received a high-fat diet. The study showed that RS2 exhibited an effect on reducing the amount of *Lachnoclostridium*, *Oscillibacter*, *Alistipes*, and *Helicobacteria*, among others—bacteria that are responsible for the cause of inflammation and are involved in the aging process [95]. Ren et al. came to similar conclusions by studying the effects of Arenga pinnata starch (APS), Arenga pinnata retrograded starch (APRS), and whole Arenga pinnata flour (APF) on intestinal microflora in aged mice. They observed, after exposure to each of the compounds tested, that the expression of p53 gene mRNA decreased and the expression of Sirt1 increased, which may indicate an “anti-aging” effect of starch [96]. Researchers also point to other benefits associated with the introduction of resistant starch into the diet. Keenan et al. point out that resistant starch plays a very important role not only in intestinal health but also in insulin resistance and obesity. Equal mechanisms are involved in increasing insulin sensitivity, including increased levels of glucagon-like peptide 1 (GLP-1) but also intestinal gluconeogenesis [97]. Resistance potato starch (RPS) has also been shown in animal models to increase the normal function of the mucosal barrier and commensal microorganisms [98]. Furthermore, the proportion of starch in the diet can reduce intestinal epithelial cell apoptosis [99]. Resistant starch has also been shown to have the ability to lower pH in the large intestine and therefore may reduce the risk of proliferation of pathogenic microorganisms [100].

Shen et al. concluded in their meta-analysis that resistant starch has a positive effect on colonic function [101]. One of the few human studies indicates that RS contributes to the maintenance of clinical remission in patients with IBD and reduces the intensity of symptoms associated with active disease. It is also associated with an increase in SCFA production [102]. In another study, Rose et al. presented that starch-entrapped microspheres, due to their maintenance of low pH and their involvement in butyrate production, can reduce harmful bacteria, for example *Bacteroides* and *Fusobacterium* [103]. The results of interviews with CD patients who had consumed wheat bran in the last 4 weeks showed a reduction in abdominal pain, cramps, and diarrhea [104].

### 3.4. Inulin

Another component of dietary fiber with beneficial effects on IBD is inulin. It is an indigestible carbohydrate that belongs to the fructan group. Some of the main sources of inulin in foods are chicory, artichokes, garlic, asparagus, and barley [105]. This compound, due to its prebiotic effect, shows the ability to change the intestinal microflora to a favorable one, such as the growth of *Bifidobacterium*. Liu et al. investigated the effect of *L. rhamnosus* 1.0320-supplied inulin on intestinal inflammation in DSS-induced animal models. They observed that the mixture showed the ability to reduce inflammatory activity and regulate the expression of pro-inflammatory cytokines, e.g., IL-1β and TNF-α. The authors also noted that inflammation caused adverse changes in the intestinal microflora, both in its composition and in the number of commensal bacteria, so the studied ingredients appear to be desirable for rebuilding a normal intestinal microflora [106]. Furthermore, a recent study showed that inulin has a regulatory effect on the microbiota in mice with DSS-induced colitis. Furthermore, it significantly reduced IL-1β and TNF-α [107]. Inulin also shows beneficial effects on intestinal barrier function in mice [108,109]. Prebiotics, including inulin, may be useful in IBD to support the therapeutic process. They show beneficial effects not only by changing the intestinal microflora to the desired ones but also indirectly promoting SCFA production. This is observed in preclinical and clinical studies [110]. In addition, inulin has been found to have a protective effect against colon cancer in mice [111].

Some authors also indicate that inulin, as a non-digestible oligosaccharide (NDO), could potentially be used as an immunomodulator in the treatment of patients with IBD [112]. Casellas et al. showed that patients with active UC had reduced fecal calprotectin levels, as well as relief from dyspeptic symptoms, after taking a 7-day supplement of oligofructose-enriched inulin. This study was well tolerated by patients and it appears that oligofructose-enriched inulin may be an effective support to reduce intestinal inflammation in patients undergoing active UC [113]. In another study, inulin was shown to modulate the gut microbiota of patients by increasing the number of bifidobacteria by 2–3 times [114]. Inulin is also increasingly being used to produce drugs with applications in IBD [115,116].

### 3.5. Fructooligosaccharides

Fructooligosaccharides are prebiotic fibers that are used most frequently in the food industry [117]. In rat studies, oral FOS supplementation was found to have a beneficial effect on intestinal inflammation. Reduce anorexia and weight loss associated with inflammation. In addition, it promotes the healing of the intestinal epithelium [118]. Another study shows that fructooligosaccharides increased SCFA (butyrate) production, decreased pH, and stimulated lactate production in mice with TNBS-induced colitis [118]. Kim et al. showed that FOS can be an effective strategy to increase *F. prausnitzii* colonization, butyrate production, and alleviate symptoms associated with DSS-induced colitis in mice [119]. Increased *Bifidobacteria* and *Enterobacteriaceae* were also observed in rats after FOS supplementation with inulin [120].

Another study involving 303 patients, which were divided into groups with active CD, inactive CD, and a group of healthy subjects, compared the common intake of fructans and oligofructose. Analysis shows that patients with active CD consumed significantly less fructans and oligosaccharides compared to other groups. This is especially observed in those with severe abdominal pain, increased CRP, and worse mood [121]. In one study, 10 CD patients received 15 g of fructooligosaccharides (FOS) for 3 weeks. FOS reduced the Harvey–Bradshaw index, and there was a significant increase in fecal *bifidobacteria* [122]. On the other hand, Benjamin et al. studied supplementation with 15 g/day of FOS in 103 patients with active CD. The patients were divided into two groups: FOS supplementation and placebo. The analysis of the results after 4 weeks did not show significant differences in clinical response between the groups [123]. Caviglia et al. investigated the effectiveness of a mixture of calcium butyrate, *Bifidobaterium bifidum*, *Bifidobacterium lactis*, and fructooligosaccharides in prolonging remission in 21 UC patients who received mesalazine (5-ASA). They showed that mixture supplementation resulted in prolonged remission in patients compared to the 5-ASA-only group. Improvements in quality of life, abdominal pain, and stool consistency were also observed, as well as in inflammatory markers [124].

### 3.6. Hemicellulose

Hemicellulose is found in germinated barley fiber (GBF), among others. A study involving rats with experimentally induced UC shows that intestinal mucosal damage and associated bloody diarrhea are prevented by using GBF in the diet of rats. GBF was shown to significantly increase butyric and acetic acid levels. Furthermore, a tendency was observed to increase the number of *Bifidobacteria* and *Eubacteria* [125].

Oligosaccharides, which are derived from hemicellulose, namely xyloligosaccharides (XOS) and mannooligosaccharides (MOS), are probiotics that are increasingly popular on the market but also manifest bioactive properties similar to FOS [126]. XOS has been shown to reduce intestinal inflammation by reducing the ratio of *Firmicutes*/*Bacteroidetes* and *Enterobacteriaceae* in obese rats [127]. MOS, on the other hand, reduces inflammation by reducing *Clostridium* in the microbiota of piglets [128].

There is a study involving patients with mild to moderate UC who received 30 g of GBF three times a day for 4 weeks. The analysis of the results showed that GBF supplementation reduced intestinal inflammation. GBF also showed a strong supportive effect on colon epithelial reconstruction. Despite continuing traditional UC treatment, patients experienced an exacerbation 4 weeks after discontinuing GBF [129]. In contrast, Kanauchi et al. showed that the use of GBF at 4 weeks significantly reduced clinical activity parameters in patients with UC [130]. A study found that hemicellulose influences a selective advantage in the human microflora but also increases protection against noncommunicable diseases [5].

The components of the fiber diet and their biological functions are shown in Table 1.

Intake of fiber in the diet provides many health benefits. Natural sources of fiber contribute minerals, vitamins, water, and a variety of phytonutrients. However, dietary fiber is not only natural food sources but also dietary supplements. According to the latest guidelines, fiber supplementation is indicated as first-line treatment for chronic constipation [131]. One of the studies shows that fiber supplementation effectively relieves constipation. In particular, psyllium, doses > 10 g/d, and treatment durations of at least 4 weeks seem optimal, although caution should be exercised in interpreting the results due to the significant heterogeneity of the study group [132].

Fiber supplements can play an important role in helping fiber intake reach recommended guidance levels. The available clinical trial data suggest that the use of fiber supplements is more effective than the use of high-fiber foods to improve serum lipoprotein values, improve weight loss, and improve gastrointestinal function. This may be due to the fact that it is easier to use tablets or powders as a secondary source of fiber than to implement proper eating practices [133]. A study indicates that whole-fiber high-fiber diet interventions resulted in more beneficial microbiome outcomes compared to low-fiber diets and fiber supplements [134]. Fiber supplementation causes moderate gastrointestinal side effects, such as flatulence, bloating, diarrhea, and abdominal discomfort, which were significantly higher with fiber supplementation compared to placebo. A review suggests that medical workers should continue to recommend that their patients eat high-fiber foods, such as fruits, vegetables, whole grains, and nuts. Possibly supplement their diet with functional fibers, such as psyllium or β-glucan. This is especially relevant as the mean dietary fiber intake by adults in the USA, it is only 17 g/d, which is clearly under the recommendation of 14 g/1000 kcal or 25 to 38 g/d, as recommended by the National Academy of Medicine [135].

## 4. Limitations

A limitation of the review may be that there are too few human and animal studies on the effects of selected components of dietary fiber on specific bacterial strains in intestinal microflora and on the effects of individual components in exacerbation and in remission in patients with IBD. Additionally, studies very often lack homogeneous groups of patients in terms of gender, age, or drugs used. However, in the above review, we selected the most reliable research and articles.

## 5. Summary

Due to one of the etiological factors of IBD, which is the alteration of the intestinal microbiota, care must be taken to ensure an adequate diet, both during the exacerbation and remission of the disease. One of the main dietary components that have a beneficial effect on the intestinal microbiota is dietary fiber. As a result of the range of action of this component, it is divided into insoluble and soluble. Increasingly, researchers are focusing on studying specific components of dietary fiber—β-glucan, pectin, starch, inulin, fructooligosaccharides, or hemicellulose—due to their individual effects in the context of IBD. Dietary fiber has been suggested to be important in the prevention of IBD by reducing pro-inflammatory cytokines, modulating the intestinal microbiota, and reducing gastrointestinal side effects. The introduction of dietary fiber in patients with IBD in remission or exacerbation should be individualized according to the individual needs and digestive capacity of the body. However, research on the properties of various components of dietary fiber and their therapeutic potential is still ongoing.

## Figures and Tables

**Table 1:** Components of the fiber diet and their biological roles [76,77,78,79,80,81,82,83,84,85,86,87,88,89,90,91,92,93,94,95,96,97,98,99,100,101,102,103,104,105].

Component	Biological Role
β-glucan	Reduction of CRP (C reactive protein) in patients with UC (ulcerative colitis). Improvement in gastrointestinal symptoms in patients taking mesalazine. Control of lipid and carbohydrate metabolism. Reduction of pro-inflammatory cytokine production. Modulation of the intestinal microbiota.
Pectin	Preventive effect of IBD (inflammatory bowel diseases).
Starch	Maintain clinical remission in patients with IBD. Reduction of symptoms associated with active disease. Increase in the production of SCFA (short-chain fatty acids) production. Reduction of inflammation in the colon. Reduction of harmful bacteria.
Inulin	Positive effect on the intestinal microbiota by increasing *Bifidobacteria*. Reduction of inflammation. Decrease in fecal calprotectin concentration. Alleviation of dyspeptic symptoms. Indirect production of SCFAs. Potential immunomodulator in IBD.
Fructooligosaccharides	Growth of fecal *Bifidobacteria*. Reduction of inflammation, indirectly affecting the reduction of anorexia and weight loss Reduction in the Harvey–Bradshaw index. Improving quality of life by reducing pain and improving stool consistency. Promoting the healing of the intestinal epithelium.
Hemicellulose	Reduction of inflammation in the intestines. Assist in the reconstruction of the intestinal epithelium. Increase the production of butyric acid and acetic acid. Increasing the number of bifidobacteria and eubacteria. Reduction in the level of clinical activity of patients with UC.

## Data Availability

Not applicable.
